# A Scoping Review on Resources, Tools, and Programs to Support Women’s Leadership in Global Health: What Is Available, What Works, and How Do We Know?

**DOI:** 10.5334/aogh.3921

**Published:** 2023-04-20

**Authors:** Eumihn Chung, Amena El-Harakeh, Jennifer L. Weinberg, Olumayowa Azeez, Ana Ortigoza, Angela Johnson, Meagan Harrison, Anna Kalbarczyk

**Affiliations:** 1 Department of Epidemiology, Johns Hopkins Bloomberg School of Public Health, Baltimore, MD, USA; 2 City University of New York, Graduate School of Public Health and Health Policy, New York, NY, USA; 3 Department of Nursing, Department of Health Sciences, Monmouth University, West Long Branch, NJ, USA; 4 National Institute of Allergy and Infectious Diseases, National Institutes of Health, Bethesda, MD, USA; 5 Urban Health Collaborative, Drexel University, 3600 Market St, 7th floor, Philadelphia, PA 19104, USA; 6 Department of International Health, Johns Hopkins Bloomberg School of Public Health, Baltimore, MD, USA

**Keywords:** women’s leadership, global health, training

## Abstract

**Background:**

The unequal representation of women in global health leadership is a prevalent issue laterally across global health fields and vertically down experience levels. Although women compose much of the workforce, gender-based barriers prevent female talent from filling their appropriate leadership roles, which funnels unique expertise and problem-solving skills on a diversity of health topics out of positions of leadership. Currently, many calls to action have been proposed to raise awareness of the lack of women’s global health leadership, with Women in Global Health as one of the more prominent movements. This paper evaluates how the priorities and strategies for leadership training and development set forth by such movements have changed the landscape of available programs and resources for women in global health, based on availability, success, and evaluation.

**Objectives:**

This manuscript maps existing programs and resources that support women’s leadership in global health and describes available evaluations and documented outcomes.

**Methods:**

We used a dual approach of a peer-reviewed and gray literature search to build a comprehensive list of existing programs and resources designed to support women’s leadership in global health. Out of 54 items included for full-text review and 22 gray literature items screened for inclusion, a total of 31 resources were processed in the final extraction. We used descriptive quantitative analysis for categorical and binary variables, while qualitative data from evaluations were analyzed for outcomes.

**Findings:**

Resources were in the form of conferences, supplemental resources to conferences, certificate programs, coursework, stand-alone documents, single-focus programs, and mostly multicomponent programs. Most resources did not have a global health focus area, and a third of the total resources identified women first authors from predominantly high-income countries. About half of the resources mention mentorship and networking as activities incorporated as part of the resource. Over half of the resources did not have a target audience, and most resources were free to users.

While there is a lack of consistent and meaningful evaluation of the resources, the available captured metrics of success were described as the number of career-advancing opportunities after using the resources. Examples of opportunities include enrollment in graduate school, receiving academic promotions, participating in internships, presenting at conferences, and publications.

**Conclusion:**

While the supply of existing programming and resources to advance women’s leadership in the global health field is limited in terms of quantity, it is rich in diverse formats, content, and implementation. This scoping review supports the notion that empowered female leadership in global health requires a complementary support system that encourages the unique needs and talents of female leaders. Such a support system needs inclusive targeting regardless of experience level, academic degree, or location. Furthermore, evaluations of resources will be critical in maintaining meaningful interventions that effectively dismantle the infrastructures that continue to limit the success of women leaders in global health.

## Introduction

Women are unequally represented in global health leadership across all sectors and especially experience inequitable gaps in obtainment and advancement in positions of leadership. While 70% percent of the global health workforce is women, as of 2018, 69% of global health organizations and 69% of ministries of health were led by men. Eighty percent of global health board chairs were men, and shockingly, in 2022, the World Health Organization’s (WHO) executive board was 91% men [1,2]. This disparity is not only demonstrated in global health organizations but also in the academic institutions in which health and global health are taught [3]. In 2016, 7 of the deans at the top 10 globally ranked schools of public health were men, and in 2018, only 18% of the deans of schools of medicine were women [4]. This gender leadership disparity occurs in health at all levels, from global health organizations to community groups [2]. As summarized by the WHO task force on the global health and social workforce, “Women deliver global health, men lead it” [2].

The lack of women in leadership is related to complex and intertwined factors and has been described as a leaky pipeline or a glass obstacle course [5,6]. The barriers preventing women from obtaining leadership positions are many and include, but are not limited to, gender biases, stereotypes, discrimination (both overt and covert), hostile work environments, power imbalances, sexual harassment, and reduced access to networks and mentors. These factors are exacerbated for women of color and women in minority groups [2,7,8,9,10].

The absence of women in positions of leadership has detrimental consequences for the entire population. Leaving women out of leadership unjustly limits diversity of perspectives and unique considerations that women bring to decision-making tables [3,9]. For example, women in leadership tend to fund educational and health programs more than their male counterparts and prioritize the needs of women, children, and marginalized groups [11]. Women leaders also give greater emphasis to certain health topics, such as reproductive rights, that benefit individuals of all genders but negatively impact women’s health more than men’s when absent [2].

Investing in women and their leadership potential can improve health for all at the individual, institutional, and community levels. It is imperative to increase the representation of women in leadership to improve the health of individuals around the world. Gender equity in global health leadership is essential to adequately addressing global health issues and especially those disparities that impact vulnerable populations across the globe.

In response to the dearth of women in global health leadership, a multitude of recommendations and calls to action have been proposed [1,5,7,8]. Women in Global Health, a movement dedicated to achieving gender parity in global health, set out five priorities to increase female representation in leadership: (1) leadership, (2) capacity building, (3) enabling environments, (4) mentoring and networking, and (5) research and data [8]. In 2021, Mousa et al. conducted a systematic review and meta-analysis on female leadership and identified five concrete strategies to increase leadership for women in all sectors, including global health. These areas include (1) organizational processes, (2) awareness and engagement, (3) mentoring and networking, (4) support tools, and (5) leader training and development [7,8].

While these priorities and strategies for leadership training and development have been well described, it is less clear what programs, resources, toolkits, and interventions currently exist or have previously been implemented. Additionally, for the efforts that have been documented in the literature, it is unclear which have been evaluated for their impact on increasing women’s representation in global health leadership. To address these gaps and identify tools, resources, and interventions for women leaders in global health, we conducted a scoping review of the peer-reviewed and gray literature [12]. This approach allowed us to quantify the volume of the body of literature and provide an overview of the current resources in this specific domain of leadership training, women, and global health [13].

This manuscript seeks to map existing programs and resources that support women’s leadership in global health and to describe the efforts that conducted evaluations and documented outcomes. Additionally, we identify areas of improvement to expand access to and success of such programs.

## Methods

We conducted a scoping review of the peer-reviewed and gray literature. We followed the Preferred Reporting Items for Systematic Reviews and Meta-Analyses Extension for Scoping Reviews (PRISMA-ScR) checklist [14]. We registered the protocol on Open Science Framework on November 6, 2021.

### Search strategy

A search strategy for the peer-reviewed literature was developed in consultation with a Johns Hopkins Bloomberg School of Public Health (JHSPH) informationist around three core concepts: (1) leadership resource, training, and/or intervention; (2) an explicit focus on women; and (3) global health. The search was conducted on October 28, 2021, in five databases (PubMed, Embase, SCOPUS, ERIC, and Business Source Ultimate) to capture literature in the global health and business and management sectors. We did not restrict articles by publication date or language.

We also conducted an iterative review of references cited in the peer-reviewed literature to identify possibly eligible gray literature resources. Then, we searched websites of relevant organizations using the specified search terms “women global leadership program” and “women global leadership initiative.”

### Study selection and eligibility criteria

A total of 2,691 references were identified and imported to Endnote for deduplication. Of these 253 duplicated articles were removed; the remaining articles (n = 2,438) were imported to Covidence, an online tool to support systematic review management, for title and abstract screening [15].

Two independent reviewers screened the titles and abstracts before completing full-text reviews for sources short-listed by at least one reviewer. Any conflicts were resolved by a third independent reviewer. Articles were included if they described a leadership resource, tool, or intervention; had a focus on women; and were explicitly related to global health or a global health issue. Global health work is a broad concept, and we landed on the definition of global health work as efforts attempting to improve the health of any population globally [16]. Articles were excluded if they did not address all three concepts and if no full text was available.

The gray literature resources were screened by one reviewer, and those that were thoroughly described by peer-review articles or did not satisfy the eligibility criteria were excluded. Screening results were verified by another experienced reviewer.

### Data charting and analysis

We developed and pilot-tested a standardized data extraction form in Microsoft Excel. Teams of two independent reviewers extracted data from each article, and conflicts were resolved by a third reviewer. The extracted data were organized into key variables that included the article’s key research questions, objectives, and sources of funding. We captured information about the leadership resources, tools, and interventions, including global health focus areas, geographic location, length of resource, advertisement of resource, target audience, form of the resource, payment information, presence of mentorship, group networking, or networking, presence of training and incorporated topics, target audiences, and any associated evaluation data, if available. Data were reviewed for discrepancies or missing data. For example, data coded as “N/A” were examined in further detail to determine whether the information for a particular variable was truly unavailable or should be coded as “no” or another category.

We synthesized the data in both narrative and tabular formats and selected appropriate variables in the final dataset to be transformed from full-text character-type data into categorical data types with unique levels. Finally, we discussed the categorical coding for each of the questions after the initial full-text extractions to create more informative variable levels.

We used RStudio to generate summary tables of the cleaned data set, including the gray literature and peer-reviewed articles. Relevant R packages used include the readr package to read in the exported dataset into RStudio and the flextable package to create the descriptive tables.

## Results

**Figure 1:**
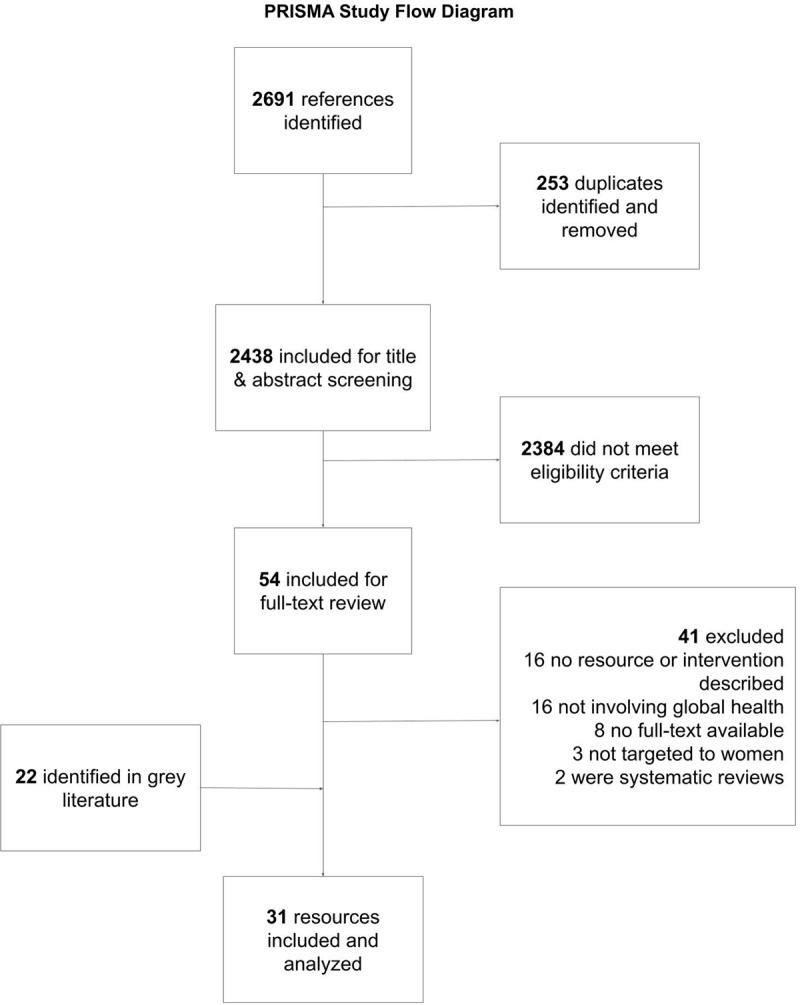
PRISMA study flow diagram.

We identified 31 articles across the published peer-reviewed (n = 9) and gray literature (n = 22) that described resources, programs, interventions, and tools for women’s leadership in global health. For simplicity, we refer to these efforts as “resources” throughout this paper. Figure 1 shows the PRISMA flow diagram of the selection process.

### Description of resources

The resources included conferences (n = 4, 12.90%) [17,18,19,20]; supplemental resource to a conference (n = 1, 3.23%) [21]; a certificate program (n = 1, 3.23%) [22]; coursework (n = 2, 6.45%) [23,24,25]; stand-alone documents (n = 8, 25.81%) [8,25,26,27,28,29,30,31]; single-focus programs (n = 1, 3.23%); and multicomponent programs (n = 14, 45.16%). Most resources (n = 20, 64.52%) did not have a specific global health focus area. Ten resources (32.23%) identified women first authors [3,8,9,27,30,31,32,33,34,35], and of those, nine (29.03%) were also affiliated with institutions in high-income countries [3,8,9,27,30,31,32,33,34,35]. As far as time commitments are concerned, most resources were not time bound (n = 18, 58.06%). Those with specific time commitments ranged from less than one week (n = 3, 9.68%) to more than one year (n = 3, 9.68%) (Table 1).

**Table 1:** Characteristics of resources.


VARIABLE N (%)	N (%)

*Format of resource*	

*Conferences*	*4 (12.90)*

*Certificate program*	*1 (3.23)*

*Coursework*	*2 (6.45)*

*Document*	*8 (25.81)*

*Supplemental resource for conference*	*1 (3.23)*

*Single focus program*	*1 (3.23)*

*Multicomponent program*	*14 (45.16)*

*Is there a mentorship component?*	

*No*	*17 (54.84)*

*Yes*	*14 (45.16)*

*Is there a group mentorship component?*	

*No*	*26 (83.87)*

*Yes*	*5 (16.13)*

*Is there a networking component?*	

*No*	*17 (54.84)*

*Yes*	*14 (45.16)*

*Length of resource, if time-bound?*	

<1 week	*3 (9.68)*

1 week	*1 (3.23)*

<1 month	*1 (3.23)*

<1 year	*2 (6.45)*

1 year	*3 (9.68)*

> 1 year	*3 (9.68)*

Not available	*18 (58.06)*

*Can the program adapt to an all-virtual model, or was it already an online resource?*	

*No*	*1 (3.23)*

*Yes*	*14 (45.16)*

*Funding sources*	

Non-profit	*16 (51.61)*

For-profit organization	*5 (16.13)*

Private academic organization	*3 (9.68)*

Public academic organization	*1 (3.23)*

NGO	*8 (25.81)*

Government	*9 (29.03)*

Other	*2 (6.45)*

Not available	*2 (6.45)*

*Author information*	

*Gender of author of resource*	*7 (22.58)*

Female	*10 (32.26)*

Male	*21 (67.74)*

*Location of authors of resource, among the female authors (n = 10)*	

HIC	*9 (90.00)*

LMIC	*1 (10.00)*

Was there a payment required for the participants in the intervention?	

No (free)	*9 (29.03)*

Yes	*4 (12.90)*

Not available	*18 (58.06)*

*If there was a payment required, how much in US dollars?*	

*$500–$800 USD*	*1 (3.23)*

*Payment required, but cost is not available*	*3 (9.68)*

*No payment required*	*8 (25.81)*

*Not available*	*19 (61.29)*

*Target audience for program: location	

*Africa Region*	*8 (25.81)*

*Region of the Americas*	*5 (16.13)*

*South-East Asian Region*	*4 (12.90)*

*European Region*	*3 (9.68)*

*Western Pacific Region*	*1 (3.23)*

*No target location*	*16 (51.60)*

*Target audience for program: only LMICs?*	

*No*	*22 (70.97)*

*Yes*	*6 (19.35)*

*Not available*	*3 (9.68)*

*Target audience for program: gender*	

*Female*	*20 (64.52)*

*No target gender*	*11 (35.48)*

*Target audience for program: degree level	

*Undergraduate*	*4 (12.90)*

*Graduate*	*4 (12.90)*

*Post-graduate*	*3 (9.68)*

*No target degree level*	*24 (77.42)*

*Programs that were inclusive of all degree levels (undergraduate, graduate, post-graduate)*	*1 (3.23)*

*Target audience for program: years of experience	

*Early career (5 years)*	*9 (29.03)*

*Mid career (5–10 years)*	*8 (25.81)*

*Late career (10+ years)*	*5 (16.13)*

*No target years of experience*	*17 (54.84)*

*Programs that were inclusive of all experience levels (early career, mid career, and late career)*	*1 (3.23)*


*these variables were select all that apply, so total counts for this variable do not sum to n = 31.

Conferences included the Women’s Federation for World Peace International First Ladies and Emerging Leaders: The Journey of 1325 & Women’s Leadership Conference [17]; the Women Leaders in Global Health conference organized by WomenLift Health [18]; the Gender Summit annual conference coordinated as part of a European Commission Project, genSET [19]; and the Women Deliver conference [20].

The one certificate program was based in Rutgers University’s Department of Women’s and Gender Studies, in collaboration with the Institute for Women’s Leadership and the National Nurses United. It offers a certificate in women’s global health leadership to individuals who complete the program [22].

Other coursework included the Essential Skills for Women’s Leadership in Global Health course, based in the JHSPH, which covered a range of topics, including understanding barriers, fostering solutions, and creating strategies for individuals and institutions to promote women’s leadership [23]. In addition, members of the Women Deliver Young Leaders Program can take online courses on topics such as gender equality and sustainable development, grant proposals and financial stewardship, and sexual and reproductive health and rights. Participants can further learn advocacy and project management through the Women Deliver’s Digital University [24].

We found one single-focus program called the Alliance-HSG Publication Mentorship Program, a six-month program for women working on health policy and systems research (HPSR) [9], but most resources (n = 14, 45.16%) featured multiple components [30,31,32,33,34,35,36,37,38,39,40,41,42,43,44].

Examples of multicomponent resources include Emerging Women Leaders in Global Health, based out of the Johns Hopkins University Center for Global Health [31,32,33,34,35,36,37,38,39,40,41,42,43]; Women in Global Health [44]; WomenLift Health: The Leadership Journey [31,32,33,34,35,36,37,38,39,40,41,42,43],; the TDR’s Women in Science Fellowship [38]; the Harvard LEAD Fellowship [39]; Building Interdisciplinary Research Careers in Women’s Health [40]; the Global Women’s Leadership Project [41]; the United Nations’ Ahfad University for Women [42]; the Moremi Initiative [43]; the Female Global Scholars Program [32]; the World Academy for the Future of Women [33]; the Higher Institute for Growth in Health Research for Women (HIGHER Women) consortium [34]; and the Malawi-Penn Women for Women’s Health project [35]. See the supplementary material for detailed descriptions of each resource.

### Common resource components

While the formats, structures, and contents of the resources varied widely, networking and mentorship emerged as common components of these resources.

#### Mentorship

Of the 31 resources included, 14 (45.16%) described mentorship as an activity, and 5 resources (16.13%) specified using a group mentorship approach [3,32,34,35,37]. Two resources (6.45%) stated that their group mentorship structure was one mentor to several mentees, grouped based on similar backgrounds and professional track [34,37]. Two resources (6.45%) that cited group mentorship were organized based on project topics [3,35], while one resource (3.23%) was categorized according to the discretion of one senior scholar [32]. Discussion topics in the group mentorship component explored leadership frameworks [37], proposal writing and workshops [34], and building collaborations [3].

Three resources (12.90%) specified mentorship models with senior mentors, such as an advisor or supervisor [32,34,40]. Two of these (6.45%) selected mentors external to the resource organization [32,34]. One resource (3.23%) described peer-to-peer mentorship in which junior scholars were paired together within similar geographic regions or research backgrounds [32]. Two resources (6.45%) required internal approval of the selected mentors: mentors were chosen from a preapproved list [40] or were selected through internal alumni relationships [28]. The TDR science fellowship had different mentorship approaches based on the location: interventions varied, such as a structured mentor/protégé program in Cameroon, peer-to-peer mentoring in Guinea, and other unspecified general mentorship methods [38].

#### Networking

Fourteen resources (45.16%) incorporated networking for women’s leadership in global health [3,8,17,18,20,24,31,32,34,36,37,38,39,43,44]. Seven resources (22.58%) included networking at an event, whether in-person or virtual event [17,18,20,32,36,39,43]. Of these events, two resources (6.45%) used an asynchronous online network or communication exchange after an event, such as using Slack [36], online messaging, or exchanging email contact information [32]. Two resources (6.45%) that included networking had varying formats, depending on the leadership of regional chapters and location [38,44]. Two resources (6.45%) that practiced networking were linked to existing mentorship programs [34,38]. Four resources (12.90%) stated that networking was included in their programming but did not specify the approach or how it was facilitated [3,8,31,37].

### Targeting

Over half of the resources did not have a target audience (n = 17, 54.84%); nine (29.03%) targeted early career professionals (5 years of experience or less), eight (25.81%) targeted midcareer professionals (5–10 years of experience), five (16.13%) targeted late career professionals, and one (3.23%) was inclusive of all experience levels. Most targeted women specifically (n = 20, 64.52%), and roughly half (n = 15, 48.39%) targeted people in specific geographic locations. Six of those with geographic target audiences were only available to participants from low- and middle-income countries (LMICs). Most (n = 20, 64.52%) did not focus on a specific global health topic area.

### Program funding and costs to participate

Most resources (n = 27, 87.00%) were free to users; however, four (12.91%) required payment for subscription or participation. One resource published its cost on a sliding scale of US$500–$800, while the other three did not indicate the specific costs to participants.

The resources were funded by the nongovernmental organizations (NGOs) implementing the resource (n = 9, 29.03), by public academic organizations (n = 8, 25.81), or by unspecified sources (n = 7, 22.58). Other sources of funding include nonprofit organizations (n = 5, 16.13%), for-profit organizations (n = 3, 9.68%), government (n = 2, 6.45%), private academic organizations (n = 1, 3.23%), or another source (n = 2, 6.45%).

### Assessment/evaluation

Of the six resources (19.35%) that reported an evaluation, three (9.68%) conducted an evaluation outside of qualitative testimonials. Of those, the captured metrics centered around the number or percentage of participants who had a career-advancing opportunity after using the resource. Examples of career-advancing opportunities included 40% of participants enrolling into graduate school [33], 6 participants receiving academic promotions [32], 8 members participating in internships [33], 11 participants presenting at conferences [32], and 41 participants writing publications [32]. One of the resources (3.23%) used an individual interview evaluation design [32], while the other two resources (6.45%) used a survey format [27,33]. Two of the resources (6.45%) mentioned an evaluation that was not published yet [23,27].

Two resources (6.45%) used qualitative testimonials to highlight how participants have benefited from participating in the intervention [3,34]. Relevant outcomes recorded from the testimonials from the GROW model include economic resources and career advancement. Two graduates reported outcomes of presenting at conferences and publication of their proposals, and three graduates cited acceptance of tenure-track offers, securing a job postgraduation, and an improvement as a qualitative and quantitative researcher [3]. One resource (3.23%) used qualitative feedback from an evaluation of a program’s workshop and reported three outcomes of value: applying to a PhD program, acceptance into a fellowship, and presenting or participating in international conferences [34].

## Discussion

This systematic review identified 31 resources published from 1997 through 2022 that met our inclusion criteria. These papers described resources for women’s leadership in global health, such as conferences, certificate programs, coursework, documents, and programs. In our review of the literature, we found that, overall, there is much variability regarding the resources that exist for training women leaders in the global health field. We found variability in format, focus, funding mechanisms, and delivery format, among other characteristics.

### Mentorship and networking

Among the variability, however, mentorship and networking were two components that were commonly offered by the resources. This was not surprising, as mentorship and networking are important for career growth [45]. Currently, there are opportunities for women such as the annual conference held by WomenLift Health, which convenes established and emerging women leaders from around the world and across various sectors [18]. Another organization with global reach, Women in Global Health [46], is the largest network of women and allies working to confront the issues of power and privilege for gender equity in health [44]. Providing opportunities for women in global health to convene, learn, and inspire one another is one way to establish networks and mentorship and to develop a consortium of tools, support, and other means by which women can effectively progress and succeed in leadership roles.

### Targeting

Our review found that many programs for women leaders in global health were broad in scope, were not specifically targeted to a particular group of women and their needs, or did not specify an intended audience. Providing effective and acceptable programming for different populations requires acknowledging and understanding their different needs. Programs should be designed and targeted to meet the needs and desired ways of engaging specific audiences. An important early step in developing programming should be to clarify the target audience(s). Establishing a clear understanding of the target audience informs subsequent decisions about the program’s design, scope, objectives, and format [47]. This can allow for more sensitive consideration of different groups’ needs.

### Needs assessments

In our review we found that needs assessments were scarce. The EDGE program was one that documented findings from a working group that asked emerging women leaders in global health for an assessment of their needs for developing leadership skills [48]. Thoughtful and thorough assessments of leadership resources and interventions must occur more frequently to engage end users in designing programming that may affect them and to better design and deliver programs that will successfully empower women to learn and take on leadership roles in global health. Stakeholders affected by decisions, programs, and policies should be involved in and able to influence the planning, conduct, dissemination, uptake, and evaluation of programming and research [49]. Stakeholder engagement throughout the course of development, implementation, and evaluation of educational and leadership programs is necessary to ensure relevance, acceptability, and feasibility; to make sure that equity and human rights issues are taken into consideration; and to support successful outcomes and improved ongoing implementation of skills and concepts learned [50].

Women in the global health field, as constituents of these programs, must be engaged at all stages of program planning, design, and evaluation. The voices of women who will participate in the programming must be heard in a way that gives them more than token involvement; they want a meaningful sense of shared ownership in the process. End user engagement improves the relevance, transparency, and usefulness of programs, and there is also an ethical obligation to engage end users in activities that may affect them [51].

### Equity

It is important to note that in our review of the literature, we found that only 10 resources (32.23%) were written by women first authors. This is ironic, given that the articles were all related to women’s global health leadership. Additionally, when the first author’s location was specified, he or she was overwhelmingly from a high-income country; this was often the case even when the programs were targeting women from LMICs.

A paternalistic approach, where an outside force solely designs and offers leadership development programs, contributes to systemic inequities perpetuated by gender-based barriers stemming from organizational constraints and culture [7]. These are many of the same reasons women, especially women from LMICs, lack leadership opportunities to begin with [52]. At all stages of program planning, implementation, evaluation, and documentation, it is important to develop clear, aligned, and equitable expectations and goals with the input of constituents.

### Evaluation

Our review also found a lack of consistent and meaningful evaluation of enacted programming. This makes it difficult to know what resources are effective and for whom. Evaluation provides an opportunity to assess the value of a given program for the participants and determines how well it met the intended objectives. A carefully targeted evaluation process that considers the outcomes women themselves identify as most meaningful to them provides valuable feedback for shaping future programming, making improvements, and ensuring that efforts match needs [53]. This process is crucial for targeting programs to constituent-identified needs, sustainability, and scalability. Conducting an appropriate and rigorous evaluation also contributes to keeping those who design and deliver programs accountable to the participants they serve, the funding agency supporting the project, and the greater goals of global health [53].

### Funding

In our review, we found that the documented resources were mostly funded by NGOs who were implementing interventions (n = 9, 29.03%), public academic organizations (n = 8, 25.81%), or unspecified sources (n = 7, 22.58%). Interestingly, only 3.23% (n = 1) were funded by private academic organizations, 6.45% (n = 2) were funded by the government, and 9.68% (n = 3) were funded by for-profit organizations, entities that would be expected to have more disposable funds. It is time that government, private institutions, and for-profit entities substantially invested in developing and maintaining women leaders in global health.

Even though most of the students pursuing studies in global health are women, the number of women in the field tends to decrease as they advance in their careers [54,55]. This may be due to the fact that women experience particular barriers to reaching leadership positions in global health, such as balancing work and home, gender bias, and lack of mentorship. Women in LMICs face additional barriers, including lack of opportunities, safety concerns, and financial constraints, more often than their counterparts in high-income countries (HIC) [27]. As such, funding opportunities like career development scholarships and grants to cultivate women leaders, especially in LMICs, is vital. Addressing these economic barriers will require strategic approaches backed by monetary resources to preserve and develop women in the field for advancement into leadership roles.

### Sustainability

In our search, we did not find much documentation about the sustainability of the resources and programs, regarding either sustained funding or the ability for women to maintain participation in global health leadership opportunities. The resources reviewed may have had sustainability strategies, but they were not included in the articles.

Steady and specific funding is key to making women’s leadership programs sustainable. For example, the National Institutes of Health (NIH) Fogarty International Center dedicates funding for research in global health specifically targeted at women [56]. Now, women make up about 33% of the leadership of NIH institutes and centers (women lead 9 out of 27 centers). The Bill & Melinda Gates Foundation has been supporting the WomenLift Health organization in scaling up interventions to support women’s leadership in health, starting in Africa and India [57]. For sustainability, these WomenLift programs are being held in collaboration with local partners in the countries where these are conducted. One of WomenLift Health’s global leadership programs is a yearlong, fully funded leadership experience aimed at increasing the confidence, networks, and understanding of barriers against developing women leaders as well as providing peer, mentor, and coaching support for midcareer women [37]. However, it is not clear whether any evaluations of these programs have been conducted.

Further, for resources and programs to be sustainable for women to participate in, due to barriers that women face regarding work-life balance, funding opportunities should accommodate adequate protected time away from work/training responsibilities, with flexible schedules for maternity leave and caregiving responsibilities [54].

### Strengths and limitations

This scoping review is subject to certain limitations. Since the research question was focused on assessing the availability of resources for women leaders in global health, one limitation is the very nature of variability in the programs publicly available. Most programs and resources may not be well documented in published peer-reviewed papers and gray literature or may not have been available in English. Relevant resources may exist beyond the scope of public online search, as formal and informal opportunities may exist within private social networks that would not be identified in our review.

Since the methods of the gray literature search were defined by the specified Google search terms, another limitation is that the team of reviewers did not screen every item from the search results due to the incredible volume of search results. In addition, the relevancy of resources presented by Google could vary based on the reviewer’s location or language, for example.

Although there was a large team of reviewers working on full-text reviews and extraction of gray literature items, there may be some variability in the comprehension of the extraction criteria, which can reduce the reliability of the results.

While there may be some variation in the results due to a diverse team of reviewers, the extraction method was piloted, and third reviewers were used to build consensus on the results. Furthermore, the results from the scoping review were found using broad and publicly available search methods, which ensures the accessibility of finding information about the resources.

## Conclusion

The existing programming and resources for advancing women’s leadership in the global health field are limited in quantity but varied in format, content, and implementation. Both women and global health settings are diverse, requiring a well-considered and intersectional approach to empowering women leaders in global health that keeps the unique needs of women in mind and incorporates their voices into the design and delivery of programming. Truly sustainable change can only be maintained by appropriately identifying and respecting women’s viewpoints and needs and authentically empowering them to build and utilize their leadership training and essential skills in a way that is meaningful to them. Including appropriate and inclusive targeting, needs assessments, and evaluations is a path to start creating effective and equitable interventions for increasing women’s global health leadership and to overcoming barriers that limit women leaders in global health.

## Additional File

The additional file for this article can be found as follows:

10.5334/aogh.3921.s1 Appendices. Appendix A and B.
