# Supplementary material for: A Scoping Review on Resources, Tools, and Programs to Support Women’s Leadership in Global Health: What Is Available, What Works, and How Do We Know?
Source: Ann Glob Health. 2023 Apr 20;89(1):27. doi: 10.5334/aogh.3921 (PMC10120609; doi:10.5334/aogh.3921)
Supplement: Appendices. — Appendix A and B. [file agh-89-1-3921-s1.pdf]

Appendix A: Resource Overview

Authors, Year	Resource Name	Resource Description	Resource Developer(s) (i.e. who made it)	Target audience(s) (i.e. who is it for)
Women Leaders in Global Health Conference, 2022	Women Leaders in Global Health Conference	The Women Leaders in Global Health Conference is a virtual, free, two day conference with speaker events, workshops and panels and networking sessions.	WomenLift Health	This resource had no target location, no target gender, no target years of experience, and was targeted for LMICs.
WomenLift Health, 2022	WomenLift Health: The Leadership Journey	The WomenLift Health: The Leadership Journey is a fully-funded and year-long multicomponent resource, including learning opportunities, mentoring, coaching, immersive experience components, as well as a self-directed leadership project.	WomenLift Health	This resource had a target location of India, East Africa, and the United States of America and was for mid-career women leaders.
Talib et al, 2017	Women Leaders in Global Health Initiative	This resource is a document that describes the impact of the Women Leaders in Global Health Conference, which calls for the advancement of women leaders at all levels of global health activity and foster continuity of intention of female leadership at the community level, academic level, in non-profit organizations, scientific societies, boardrooms, and the government. This document informs readers of the Women in Global Health vision for gender parity of 50-50 representation in the top global health leadership positions by 2030. This document describes how global health events are held accountable to the Women Leaders in Global Health Initiative's checklist, which calls for even representation of perspectives among male and female role models.	Women in Global Health	This resource had no target location, no target gender, and was for early and mid-career professionals.
Rutgers School of Arts and Sciences Department of Women's Gender, and Sexuality Studies, 2022	Rutgers Online Certificate Program in Women's Global Health Leadership	The Rutgers Online Certificate Program in Women's Global Health Leadership supports women's leadership by providing course work that addresses pressing global health issues. This certificate program is the product of a collaboration with Rutgers Department of Women's and Gender Studies with National Nurses United, the largest nurses union in the U.S. and the Institute for Women's Leadership. Students must take a total of 15 credits of coursework, with required courses on women’s global health movements and gender, economic inequality, and health.	Collaboration between Rutgers Department of Women's and Gender studies with Institute for Women's Leadership (IWL) and National Nurses United (NNU)	This resource had no target location, no target gender, and was for undergraduate or graduate level students
Johns Hopkins Bloomberg School of Public Health, n.d.	Essential Skills for Women's Leadership in Global Health	The Essential Skills for Women's Leadership in Global Health is a one week course offered at the Johns Hopkins Bloomberg School of Public Health to develop an understanding of the barriers women face in leadership and to foster solutions and strategies for individuals and institutions. This course explores leadership frameworks and builds essential skills such as self-awareness, communication, and negotiation.	Johns Hopkins Bloomberg School of Public Health	This resource had no target location, no target gender, and was for all students (no target degree level).

Johns Hopkins University Center for Global Health, n.d.	Emerging Women Leaders in Global Health	Emerging Women Leaders in Global Health (EDGE) is an multicomponent resource that supplies women with access to resources, tools, and skills. The initiative includes a monthly seminar series, a Slack network, and a 3-credit JHSPH summer institute course.	Johns Hopkins Center for Global Health	This resource had no target audience, had a target gender of women, non-binary individuals, and men, and for women leaders in training and/or in their mid stages of career.
Special Programme for Research and Training in Tropical Disease, 2022	TDR: Women in Science	TDR: Women in Science is a multicomponent resource designed to improve the careers of women scientists in academic and research institutions in 9 countries in Sub-Saharan Africa (Cameroon, Congo, Ethiopia, Guinea, Kenya, Malawi, Mali, Nigeria, and Uganda). The fellowship included mentoring, manuscript and proposal development, networking, and research methodology and ethics skills building.	World Health Organization Special Programme for Research and Training in Tropical Diseases (TDR)	This resource had a target location of 9 countries in Sub-Saharan Africa (Cameroon, Congo, Ethiopia, Guinea, Kenya, Malawi, Mali, Nigeria, Uganda), and was for mid-career women scientists in LMICs.
Harvard Global Health Institute, 2022	Harvard LEAD Fellowship for Promoting Women in Global Health	The Harvard Lead Fellowship for Promoting Women in Global Health is a multicomponent resource designed to support leadership skills among individuals from low- and middle-income countries. Fellows are paired with senior mentors and have the opportunity to take coursework, engage in a tailored leadership training program, participate in speaking and networking opportunities, and pursue independent project work.	Harvard T.H. Chan School of Public Health	This resource had a target location of low and middle income countries, no target gender, and no target degree level. The resource states that they welcome "all continents, regions, disciplines, sectors, gender and gender identities ... with no age or academic prerequisites."
Women in Global Health, 2022	Women in Global Health	Women in Global Health is a multicomponent resource designed to foster a global network of women to organize and connect advocates for women leaders and to support a digital movement through a digital campaign around gender and health. The initiative has over 41 Women in Global Health Chapters in 36 countries.	Women in Global Health	This resource listed broad locations such as Africa, Cameroon, Canada, United States of America, Finland, Francophone, Germany, Midwest, Norway, Pakistan, Somalia, and Sweden. There is no target gender and no target degree level.
Gender Summit, 2019	Gender Summit	Gender Summit is a conference to promote gender equality in research and innovation, by increasing the number of women in scientific roles, improving the integration of sex-gender analysis in research, and supporting cross-cutting benefits of gender-sensitive and response research and innovation. Each Gender Summit conference produces documents or reports to further promote their mission to support gender equality and women's empowerment.	Gender Summit	This resource is used broadly in Europe, North America, Africa, and Asia Pacific, with a target audience of women, and no target degree level.
National Institutes of Health: Office of	Building Interdisciplinary Research Careers in	The Building Interdisciplinary Research Careers in Women's Health (BIRCWH) is a multicomponent resource that offers	National Institutes of Health: Office of	This resource had no target location, no target gender,

Research on Women's Health, n.d.	Women's Health	financial support and a mentored career-development program connecting junior faculty, in participating institutions, to senior faculty with common interests.	Research on Women's Health	and target degree level of post-graduate (recently completed clinical training or postdoctoral fellowship).
London School of Hygiene and Tropical Medicine, 2022	London School of Hygiene and Tropical Medicine: Insights from women leaders	The London School of Hygiene and Tropical Medicine: Insights from women leaders is a supplemental resource for the Women Leaders in Global Health conference, where women leaders with an association with the London School of Hygiene and Tropical Medicine share their advice for early-career women.	London School of Hygiene and Tropical Medicine	There was no target location, targeted to women, and no target degree level or years of experience.
World Health Organization, 2021	Closing the leadership gap: gender equity and leadership in the global health and care workforce	This resource is a document that serves as a policy action paper to change the legal foundation for workplace equality, address social norms/stereotypes, and acknowledge workplace systems and culture, while encouraging women in the health and social care workforce to become leaders.	World Health Organization	This resource had no target location, no target gender, with no target degree level or years of experience. The resource is stated to be broadly for national authorities and intersectional policy-makers, organizational leaders, publishers, media, male allies, and women in healthcare.
World Health Organization, 2019	WHO Global Health Workforce Network’s Gender Equity Hub: Delivered by women, led by men: a gender and equity analysis of the global health and social workforce	This resource is a document that provides a gender and equity analysis of the global health and social workforce. This report summarizes over 170 gender and equity studies in global health and argues that the loss of female talent impacts global health. Therefore, more representation of women in leadership positions could resolve more health challenges.	World Health Organization	This resource has no target location, targeted for women, with no target degree level or years of experience.
Liu et al., 2019	Women’s global health leadership in LMICs Comment - The Lancet	This resource is a document that highlights the lack of low- and middle-income voices in women's leadership in global health. This document highlights the unequal progress towards gender equality in global health institutions in high income countries compared to LMICs.	The Lancet	This resource is for LMIC's, for females, and with no target degree level or years of experience.
University of Pennsylvania Carey Law School, 2022	Global Women’s Leadership Project	The Global Women's Leadership Project is a multicomponent resource that provides research for UNESCO and UN Women to support work on women, peace, justice, and women's human rights.	University of Pennsylvania Carey Law School	This resource has no target location, for women, for graduate students, and with no target years of experience.
Women Deliver, 2021	Women Deliver: Conference	The Women Deliver: Conference connects advocates and decision makers from a variety of fields and serves as a policy moment and accountability check for the commitments made for gender equality internationally.	Women Deliver	This resource has no target location, no target gender, no target degree level, and no target years of experience.

Women Deliver, 2018	Women Deliver: Strengthening Skills	The Women Deliver's Digital University has online coursework for every Women Deliver leader. The learning experience includes peer-to-peer learning, networking with subject matter experts, advocacy, and project management. Furthermore, there are courses that apply gender lens to the UN's Sustainable Development Goals and invests in women leaders and advocates.	Women Deliver's Digital University	This resource has no target location, no target gender, no target degree level, and no target years of experience.
The George Institute, 2022	The George Institute for Global Health: Kerala Study - Women Leaders in Global Health (WLGH)	This document highlights the lack of female representation in population health, especially at higher leadership levels. The resource identifies women leaders in Kerala, India specifically to potentially raise awareness of women leaders in the health sector and their accomplishments.	The George Institute and Women Leaders in Global Health	This resource has a target location of Kerala, India, is targeted for women, with no target degree level, and is for early, mid, and late career women. This resource is for LMICs.
United Nations Academic Impact, n.d.	United Nations: Ahfad University for Women	The Ahfad University for Women is a multicomponent resource that prepares women to fulfill community leadership roles by supplying them with academic courses, job training, independent research, and community extension activities.	United Nations Academic Impact	This resource is targeted for women in Sudan, with undergraduate and graduate degrees, and has no target years of experience.
Women's Federation for World Peace International, n.d.	Women's Federation for World Peace International: First Ladies and Emerging Leaders: The Journey of 1325 & Women's Leadership	This resource is a conference with the aim to inform, engage, and inspire women's empowerment and leadership. The conference includes online programs, a forum, and more than 500 events	United Nations Commission on the Status of Women	This resource has no target location, for women, no target degree level or target years of experience.
Moremi Initiative, 2021	Moremi Initiative: Young Professional & Organisational Leadership Program - Women Leadership in Africa	The Moremi Initiative is a multicomponent resource for women's leadership in Africa, utilizing leadership development, mentorship, and research and advocacy programs and workshops to encourage female leaders in global health issues.	Moremi Initiative	This resource has no target location, for women, with no target degree level, and is for women in their mid-career and late careers.
Yount, 2018	GROW: a model for mentorship to advance women's leadership in global health	The GROW (Global Research for Women) model is a multicomponent resource that supports women's empowerment through the acquisition of human, economic, and social resources. The initiative is guided by the principle that women's empowerment is an integral part of sustainable development and can be achieved by advancing scholarship, cultivating leadership through practicums, group mentorship and conferences, and networking.	data from pilot study from Emory University	This resource has a target location of the United States, has no target gender, for undergraduate, graduate, postgraduate degree levels and for early career, mid career individuals.
Walsh, 2021	Novel, Low-Cost Intervention to Promote Women's Advancement in Global Health Research	The resource is a multicomponent resource, known as the Female Global Scholars Program in the Weill Cornell School of Medicine. This program offers leadership skill training, peer mentoring for global health research, workshops, webinars, and group mentorship. The paper describes the success of the inaugural cohort of the program, measured by academic	Weill Cornell School of Medicine	This resource is targeted for women in Haiti, India, Kenya, Tanzania, Uganda, and the United States, has no target degree level, and is for women of early

		promotions, presentation at international conferences, and publication of abstracts and manuscripts.		careers.
Moyer, 2018	Advancing women leaders in global health: Getting to solutions	This was a document that studies the results of a convergent mixed-methods, cross-sectional, anonymous online study of participants of the Women Leaders in Global Health Conference in 2017. Proposed solutions from this study include senior women seeking mentorship relationships among junior women, and institutional intervention to incentivize mentorship to improve diverse leadership. The study explores barriers women face, as well as qualitative testimonials	Women Leaders in Global Health	This resource has no target location, is for women, has no target degree level, and is for early, mid, and late career individuals.
Martin, 2015	The World Academy for the Future of Women: A global model to women's leadership	The World Academy for the Future of Women leadership preparation program was a multicomponent resource for young women, with the goal to empower female passion and purpose in global health leadership. This paper assesses the success of the volunteer model of the WAFW, possible next steps to improve, as well as findings from completion of the program.	World Academy for the Future of Women (WAFW)	This resource is for individuals in China and LMICs generally and targeted towards women of undergraduate degree levels.
Ludwig, 2018	Women in Global Health-Germany network	This document resource describes the Women in Global Health- Germany network as an exceptional example of the broader multicomponent resource, Women in Global Health. The Germany chapter serves as a great example of increasing the number of women leadership positions within global health and highlights their accomplishments. The Germany chapter meets regularly to maintain a flexible and engaged network and hold events such as conferences.	Women in Global Health	This resource is targeted towards women in Germany and high income countries in general, with no targeted degree levels, and is for women in their late careers in global health.
KwediNolna, 2017	Mentoring for early-career women in health research: The HIGHER Women Consortium approach	The HIGHER Women consortium has a multicomponent resource known as the Mentor-Protege Program to support mentee's growth and success in health research. The program includes workshops focused on proposal writing for projects, and they have opportunities to present at international conferences.	HIGHER Women consortium	This resource is targeted towards women research scientists in Cameroon in their early careers, with no target degree level.
Javadi, 2020	Enhancing diversity in public health scholarship: the role of publication mentorship	This resource is a six-month publication mentorship structured program supporting early-career women in the field of Health Policy and Systems Research in low- and middle-income countries.	collaboration between Alliance for Health Policy and Systems Research and Health Systems Global	This resource is for women in LMICs, with no target degree level and in their early-careers.
Dhatt, 2017	The time is now - a call to action for gender equality in global health leadership	This is a document, or a call to action paper, to prioritize gender equality in global health's leadership. The goal is to reach a minimum 50-50 representation in women's leadership in global health across all key stakeholders by 2030, by ensuring each global health leader has public commitments and a plan to achieve their gender equality goals. This document has a comprehensive list of the commitments made by the global health community.	Women in Global Health	This resource has no target location, no target gender, and no target degree level or years of experience.

Brooten, 1997	Collaborating for international research development in Malawi, Africa	This is a multicomponent resource to support women leaders in Malawi to develop and conduct research on women and infant health. The 5-year program includes a workshop in research, creating and testing pilot projects, project development, and dissemination of findings. Two of the studies from this program are studied in detail, with results that suggest important health policy implications for Malawi.	Malawi Ministry of Health and University of Pennsylvania School of Nursing	This resource is for women in Malawi, with no target degree level and for women in their early careers.

Appendix B: Evaluation

Author, Year	Resource/Program Name	Type of Evaluation (i.e. study design)	Findings	Recommendations
(Yount et al. 2018)	GROW: a model for mentorship to advance women's leadership in global health	Qualitative analysis, assessing the success of a pilot study of the GROW mentorship model	This resource describes the components of the Global Research for Women (GROW) model, which is based on empowerment and advancement of women's careers in global health, and presents qualitative testimonials describing benefits of each aspect of the framework. The domains of women's empowerment explored in the model include human resources, economic resources, social resources, intrinsic agency, instrumental agency, collective agency, career advancement, and women's leadership in global health. Relevant metrics recorded within the economic resources and career advancement domains include presenting at conferences, publication of proposals, accepting tenure track offers, securing jobs post-grad, and more qualitative and quantitative experience in the field.	Authors recommend designing a supplemental program and to conduct a randomized clinical trial to compare empowerment processes.
(Walsh et al. 2022)	Novel, Low-Cost Intervention to Promote Women's Advancement in Global Health Research	Quantitative analysis, evaluating objective, quantitative data collected through individual interviews with scholars.	Quantitative success of the program is measured by the number of promotions, conference presentations, and abstract/manuscript submissions. Metrics measured at the end of year 1 include 6 participants receiving academic promotions and 10 inaugural scholars collectively presented at 11 international conferences and submitting 22 abstracts and 19 manuscripts.	Authors stated that they will conduct an in-depth evaluation and explore the program's short-term and intermediate outcomes. The evaluation at the end of the second year was incomplete at the time of publication of the paper
(Moyer et al. 2018)	Advancing women leaders in global health: Getting to solutions	A convergent, mixed-methods, cross-sectional anonymous online study.	Qualitative metrics that were recorded include how respondents ranked barriers to women's advancement in global health, as well as whether respondents believed that gender bias had impacted their own career growth in global health. Leading barriers to addressing advancement for women in global health include lack of mentorship, challenges of balancing work and home, gender bias, and lack of assertiveness/confidence.	Authors recommend future research to identify key metrics of success and to measure improvement overtime. In addition, the impact of various interventions contributing to the improvement of such measures should be further explored. An evaluation is ongoing but not published yet.
(Martin et al. 2015)	The World Academy for the Future of Women: A global model to women's	Qualitative survey	Quantitative measures recorded include the percent enrollment into graduate school (40% of participants), and the number of external internships in the World	The survey has been redesigned to allow for pre and post-test data collection from participants and

	leadership		Academy for the Future of Women (increased from 1 to 9 in two years). In addition, 90% of participants state that the program enabled them to follow their passion.	facilitators. Authors recommend matching the facilitators with the participants each year to get in-depth qualitative data overtime.
(Kwedi Nolna et al. 2017)	Mentoring for early-career women in health research	Qualitative evaluation of workshops and general feedback from program participants	Four outcomes of value were reported: applying to a PhD program, acceptance into a fellowship, and presenting or participating in international conferences.	The authors recommend motivating mentors with resources for network socials and small group workshops. In addition, the authors advise working with universities and research institutions to improve recruitment and retention of participants. Lastly, a central African forum to include networks in Gabon, Congo, Equatorial Guinea, Chad, and Central African Republic is recommended for extended networking and collaborative opportunities.
